# Functional characterisation of long intergenic non-coding RNAs through genetic interaction profiling in *Saccharomyces cerevisiae*

**DOI:** 10.1186/s12915-016-0325-7

**Published:** 2016-12-07

**Authors:** Dimitris Kyriakou, Emmanouil Stavrou, Panayiota Demosthenous, Georgia Angelidou, Bryan-Joseph San Luis, Charles Boone, Vasilis J. Promponas, Antonis Kirmizis

**Affiliations:** 1 Department of Biological Sciences, University of Cyprus, Nicosia, CY-1678 Cyprus; 2 The Donnelly Centre, University of Toronto, 160 College Street, Toronto, Ontario M5S 3E1 Canada

**Keywords:** Long intergenic non-coding RNAs, Stable unannotated transcripts, Synthetic genetic array, Genetic interactions, Telomere

## Abstract

**Background:**

Transcriptome studies have revealed that many eukaryotic genomes are pervasively transcribed producing numerous long non-coding RNAs (lncRNAs). However, only a few lncRNAs have been ascribed a cellular role thus far, with most regulating the expression of adjacent genes. Even less lncRNAs have been annotated as essential hence implying that the majority may be functionally redundant. Therefore, the function of lncRNAs could be illuminated through systematic analysis of their synthetic genetic interactions (GIs).

**Results:**

Here, we employ synthetic genetic array (SGA) in *Saccharomyces cerevisiae* to identify GIs between long intergenic non-coding RNAs (lincRNAs) and protein-coding genes. We first validate this approach by demonstrating that the telomerase RNA TLC1 displays a GI network that corresponds to its well-described function in telomere length maintenance. We subsequently performed SGA screens on a set of uncharacterised lincRNAs and uncover their connection to diverse cellular processes. One of these lincRNAs, *SUT457*, exhibits a GI profile associating it to telomere organisation and we consistently demonstrate that *SUT457* is required for telomeric overhang homeostasis through an Exo1-dependent pathway. Furthermore, the GI profile of *SUT457* is distinct from that of its neighbouring genes suggesting a function independent to its genomic location. Accordingly, we show that ectopic expression of this lincRNA suppresses telomeric overhang accumulation in *sut457Δ* cells assigning a *trans*-acting role for SUT457 in telomere biology.

**Conclusions:**

Overall, our work proposes that systematic application of this genetic approach could determine the functional significance of individual lncRNAs in yeast and other complex organisms.

**Electronic supplementary material:**

The online version of this article (doi:10.1186/s12915-016-0325-7) contains supplementary material, which is available to authorized users.

## Background

Genome-wide transcriptional studies have revealed that eukaryotic genomes are pervasively transcribed, producing thousands long (>200 nucleotides) non-coding RNAs (lncRNAs) [1]. Nevertheless, functional characterisation of lncRNAs is lagging considerably behind their discovery rate [2]. One reason for this discrepancy is the fact that only a small proportion of these molecules are essential for life as shown by knockout studies of mammalian lncRNAs [3]. Therefore, the cellular effects of a mutated lncRNA might be masked by other factors with compensatory functions, indicating that the role of lncRNAs should be interrogated under synthetic knockout conditions. Another challenge for determining lncRNA functions stems from the fact that genetic manipulations of certain lncRNAs often affect the expression of other overlapping genes [2]. This issue is, of course, less true for long intergenic non-coding RNAs (termed lincRNAs), which do not overlap any other genomic features.

In *Saccharomyces cerevisiae*, approximately 85% of the genome is transcribed, generating a large number of lncRNAs [4, 5] that are reminiscent of those found in higher eukaryotes [6]. Among these non-coding transcripts there are classes of lncRNAs sensitive to RNA decay machinery, such as cryptic unstable transcripts, Xrn1-sensitive unstable transcripts and telomeric repeat-containing RNAs [4, 7–13], as well as lncRNAs expressed only under specific conditions such as meiotic unannotated transcripts [14], cytoplasmically degraded-cryptic unstable transcripts [15], stress-inducible lncRNAs [16] and telomerase mutant lncRNAs [17]. In addition, there are stable lncRNAs in wild-type yeast appropriately defined as stable unannotated transcripts (SUTs), which represent approximately 12% of the yeast transcriptome [11]. SUTs evade degradation in the nucleus and are processed in the cytoplasm in a similar manner to mRNAs [6]; thus, it was suggested that these transcripts might be functionally important [2, 18].

Only a handful of SUTs have been experimentally investigated and most of these have been assigned roles in regulating gene expression. In fact, it is the transcriptional process of some of these characterised lncRNAs that has a functional output and not the transcripts themselves [2]. For example, transcription of the lncRNA designated as *IRT1* (*IME1* Regulatory Transcript 1, also known as *SUT643*) represses the expression of its adjacent *IME1* protein-coding gene by establishing repressive chromatin modifications at the *IME1* promoter [19]. Additionally, transcription of the *GAL10* lncRNA (aka *SUT013*) recruits similar chromatin modifying activities to alter nucleosome occupancy and to repress the expression of *GAL1* and *GAL10* [20, 21]. Likewise, the transcription of two other lncRNAs, *ICR1* and *PWR1*, modulates chromatin structure and transcription factor binding at the *FLO11* promoter [22]. Other steady-state yeast lncRNAs control gene expression through transcriptional interference and not through recruitment and modulation of chromatin modifications. For instance, transcription of the *IME4*-antisense lncRNA blocks sense strand transcription of the *IME4* gene [23] and transcription of the *SRG1* lncRNA prevents transcription initiation at the *SER3* promoter [24]. A common feature of almost all characterised yeast lncRNAs, including the SUTs described above, is that they function in *cis* by regulating their cognate genes. Therefore, it remains unclear whether a significant number of yeast lncRNAs could also function in *trans*, distant from the locus from which they are transcribed, in order to regulate gene expression and other DNA-based processes.

The RNA component of the telomerase complex, known as telomerase component 1 (TLC1) [25], constitutes the single most characterised yeast lincRNA that functions in *trans*. TLC1 has a functional orthologue in human cells, known as TERC [26], but these two RNAs vary in size and nucleotide sequence. TLC1 is transcribed on chromosome II and then interacts physically with proteins Est1, Est2 and Est3 to form the telomerase complex, whose role is to solve the end replication problem by synthesising telomeric DNA repeats and preventing telomere shortening during each cell division [27, 28]. TLC1 is recruited to telomeres through a chain of physical interactions coordinated by the heterodimeric Ku complex (yKu70/yKu80) [29–31]. Once at the telomeres, TLC1 functions as the template for telomere DNA synthesis by Est2, the reverse transcriptase component of the telomerase complex [27]. In the absence of functional telomerase, telomeres are significantly shortened and cells stop dividing and senesce [32]. Within a population of senescing cells some survivors arise by fixing their telomere length through a DNA recombination-based mechanism [33]. This alternative telomere maintenance mechanism is mediated by two distinct Rad52-dependent DNA recombination pathways; a commonly induced Type I pathway, which requires the Rad51, Rad54, Rad55 and Rad57 proteins, or a Type II pathway, which involves the trimeric MRX complex (consisting of Mre11, Rad50 and Xrs2) and Rad59 [33, 34]. Additional proteins, including the Ino80 chromatin remodelling complex, function as regulators of the telomere recombination mechanisms [34] in order to preserve telomere length and function.

Telomere homeostasis is also dependent on the presence of 3’-end single stranded DNA (ssDNA) at chromosome ends, known as telomeric overhangs. In most organisms, the telomeric overhangs consist of G-rich repeats and extend over the C-rich strands at both ends of a chromosome [27]. Telomeric overhangs are necessary during telomere replication because they provide a substrate for the RNA moiety of the telomerase complex. Formation of telomeric overhangs is closely linked to DNA replication, occurs independently of telomerase action and is regulated by evolutionary conserved mechanisms [35]. In *S. cerevisiae*, various exonucleolytic activities are involved in the formation of telomeric ssDNA, including the MRX complex and the 5’–3’ double-strand-specific exonuclease Exo1 [36]. The nuclease activity of these enzymes towards chromosome ends is inhibited by telomeric capping factors, such as Rap1, Rif1 and Rif2, that block MRX access, and the CST (Cdc13-Stn1-Ten1) and Ku complexes, which protect telomeres from Exo1-mediated degradation [37, 38]. Defects in telomere capping that can impact on the activity of exonucleases towards telomere ends lead to various problems, including premature senescence, cell-cycle arrest and accumulation of telomeric ssDNA [37, 39–41]. Hence, complete understanding of the factors and mechanisms that control telomere-end processing is important for determining how telomeres maintain their structure and function [27].

In this study, we sought to identify genetic interactions (GIs) between yeast lincRNAs and protein-coding genes in order to evaluate their functional relationships using synthetic genetic array (SGA) technology. As proof of principle, we initially demonstrated that the GI network of the lincRNA TLC1 is consistent with its function in telomere maintenance. Therefore, we next applied the same systematic genetic approach to six other uncharacterised intergenic SUTs. Interestingly, the GI profile of *SUT457* connected this lincRNA to telomere organisation and follow-up experiments established SUT457 as a novel factor of telomere overhang homeostasis. This study proposes that systematic analysis of GIs could unveil the function of lincRNAs in *S. cerevisiae* and other complex organisms.

## Results

### The GI network of *TLC1* corresponds to its cellular function

**Fig. 1:**
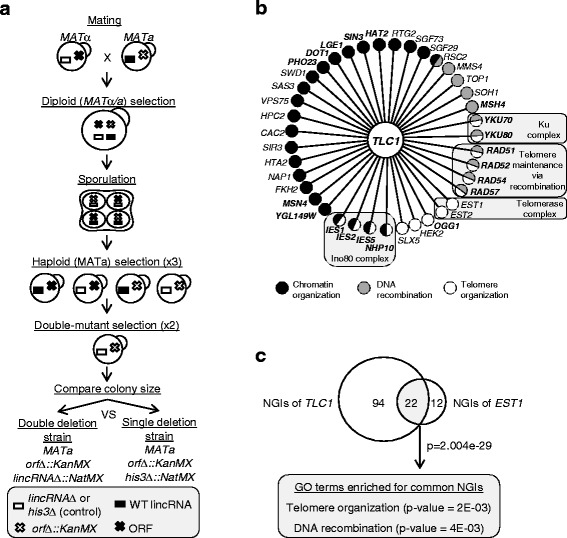
*TLC1* validates the SGA approach for interrogating the function of lincRNAs. **a** Schematic illustration of the SGA screening procedure used for studying lincRNAs. A query strain (MATα) carrying deletion of a lincRNA (white box) is mated to an array of 4309 single knockout strains (MATa), each lacking a non-essential protein-coding gene (*white* X mark). Following several selection steps, double mutant strains are isolated and their growth fitness is compared to the corresponding single deletion strains derived from a parallel control SGA screen. **b** Genetic interactions (GIs) of *TLC1* associated with the three indicated Gene Ontology (GO) biological processes. Gene nodes are coloured according to their assigned GO annotation. Negative (bold letters) and positive (regular font) GIs that belong to the three GO terms are shown. Previously established *TLC1* cofactors are highlighted in light grey boxes. **c** Venn diagram showing the significant overlap (*P* = 2 × 10^–29^) between genes classified as having a negative GI with *TLC1* and *EST1* in this study. The *P* value was generated using the hypergeometric test (phyper)

Although genome-wide transcriptional studies have revealed an enormous amount of non-coding RNAs that can be synthesised from the *S. cerevisiae* genome, a relatively small number of these lncRNAs have been functionally characterised [2, 42]. We reasoned that we could obtain insights about the function of yeast lincRNAs by comprehensively mapping their GIs through an approach that has been previously applied for protein-coding genes [43]. Therefore, we employed the SGA methodology to construct double mutants in which the deletion of an intergenic SUT (*sutΔ*) is systematically combined with individual deletions of non-essential genes in budding yeast*.* GIs are scored in double mutants that show significant deviation in fitness compared to the growth of the corresponding single gene deletion strains generated during the control SGA screen (Fig. 1a and Methods). Specifically, a negative GI (NGI) refers to a more severe fitness defect in the double mutant compared to the corresponding single gene deletion mutants, while a positive GI (PGI) corresponds to growth with a less severe fitness defect in the double mutant in comparison to the single deletion mutants. The precise stepwise procedure used for the SGA screens and the deviations from a conventional approach [44] are shown in Fig. 1a (see also Methods).

**Fig. 2:**
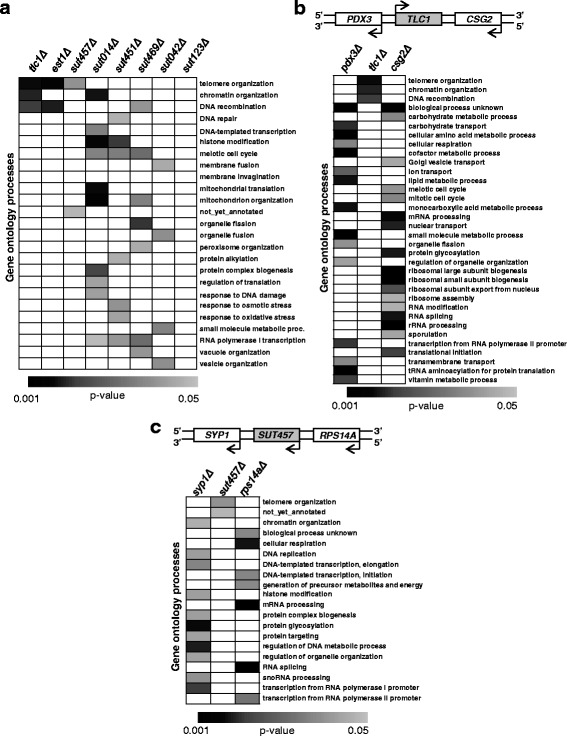
Genetic interactions link intergenic SUTs to diverse biological processes. **a** Heatmap of gene ontology (GO) terms enriched by genes whose deletions result in negative genetic interactions. The synthetic genetic array (SGA) procedure was performed for *TLC1*, *EST1* and six intergenic SUTs. The negative genetic interaction (NGIs) from the different SGA screens were analysed as described in Methods to identify enriched GO terms with *P* ≤ 0.05 (generated using Fisher exact test). **b** and **c** Heatmap of GO terms enriched by genes classified as having a NGI with *TLC1* (**b**), *SUT457* (**c**) or their corresponding flanking genes. The NGIs of the genes flanking the tested lincRNAs were extracted from the Drygin database (http://drygin.ccbr.utoronto.ca/) and Szappanos et al. [53]

To demonstrate proof of concept for the aforementioned rationale, we initially applied the SGA procedure to the yeast lincRNA *TLC1*, which has a well-defined cellular role in telomere maintenance [27, 28]. The SGA screen was carried out in duplicate by combining the *tlc1Δ* strain against an ordered array of approximately 4300 viable protein-coding gene deletion strains. A total of 116 NGIs and 261 PGIs of *TLC1* were identified within the two SGA screens (Additional file 1: Table S1 and Additional file 2: Table S2). We identified NGIs with Ku complex components (*YKU70/80*) whose synthetic lethal interactions with *TLC1* were previously reported [30]. Moreover, *TLC1* showed NGIs with genes (*RAD51*, *RAD52*, *RAD54*, *RAD57*) whose proteins are integral components of the Rad52-mediated telomere recombination pathway [33] and the Ino80 chromatin remodelling complex that is also implicated in telomere lengthening via homologous recombination [34] (Fig. 1b). Since PGIs are typically detected among components of the same protein complex [45, 46], we also found that *TLC1* has a positive GI with its co-factors *EST1* and *EST2*, which are subunits of the telomerase complex. To further support the relevance of the identified GIs with the function of *TLC1*, we then performed gene ontology (GO) analysis of our SGA data, as an objective metric for deriving functional utility from GI datasets [47]. We chose to perform GO enrichment analysis using only NGIs, since the SGA technique demonstrates higher precision rate on detecting true NGIs compared to true PGIs and, in addition, NGIs frequently occur between genes with overlapping functions [43, 46, 48]. In agreement with the individual GIs mentioned above, GO analysis of all *TLC1* NGIs significantly enriched the biological process terms, telomere organisation (*P* = 0.00081) and DNA recombination (*P* = 0.0049) (Figs. 1b and 2a). Furthermore, *TLC1* NGIs significantly enriched the term chromatin remodelling (*P* = 0.0027), a cellular activity that has been associated with telomere maintenance [34, 49–51] (Figs. 1b and 2a). These results show that the SGA-derived GIs of *TLC1* are consistent with the cellular function of this yeast lincRNA.

It was previously demonstrated that genes with highly similar GI profiles have strong functional relationship to the extent that they can be physical partners within the same complex [43]. Hence, we hypothesised that *TLC1* should have a GI profile that is highly correlated to the profile of other components of the telomerase complex. To test this hypothesis and support the validity of our *TLC1* SGA screens, we performed an independent SGA screen for the protein-coding gene *EST1*, which encodes a subunit of the telomerase complex. We identified 34 NGIs for *EST1*, of which 22 interactions were common with *TLC1* NGIs (*P* = 2.004 × 10^–29^), indicating that their GI profiles are highly related (Fig. 1c). The shared GIs between *EST1* and *TLC1* enrich for the GO terms telomere organisation and DNA recombination that are related to their cellular function (Fig. 1c). To further examine the validity of our SGA screens, we also compared our identified *TLC1* GIs to *EST1* GIs reported in previous studies. Notably, about 40% of *TLC1* NGIs identified in this study were also identified as *EST1* negative GIs by two independent SGA studies [43, 52], and once again, the common NGIs between *TLC1* and *EST1* enrich the GO terms telomere organisation and DNA recombination (Additional file 3: Figure S1a, b). Overall, these findings demonstrate that SGA-derived GIs can illuminate the biological role of *TLC1*, and support the utility of this approach in identifying the function of other uncharacterised yeast lincRNAs.

### SGA analysis implicates intergenic SUTs in diverse cellular processes

The *S. cerevisiae* genome encodes approximately 850 SUTs [11], of which 95 are intergenic (Additional file 4: Table S3) since they do not overlap any other known genomic features (Additional file 5: Table S4). We applied the SGA procedure to six intergenic SUTs that differ in size, distance from adjacent genes and level of expression [4, 11] (Additional files 4 and 6: Table S3 and S5). The SGA screen for each deleted lincRNA was paired with a control SGA screen (Fig. 1a) to minimise the ‘batch effect’ [46]. We identified in total 606 negative GIs and 1079 positive GIs for all six tested SUTs (Additional files 1 and 2: Tables S1 and S2). We subsequently analysed the NGIs of each SUT (Additional file 1: Table S1) for the enrichment of GO biological process terms as described for *TLC1* above. Notably, all tested lincRNAs enriched at least one GO term with the exception of *SUT123* which did not highlight any terms despite displaying 74 NGIs (Fig. 2a and Additional file 1: Table S1). It is possible that this lincRNA is involved in multiple processes that are diluted among the identified GIs. The other five SUTs exhibited NGIs that result in distinct GO enrichment profiles (Fig. 2a). In particular, the SGA screen using the *sut457Δ* strain enriched for the two GO terms, telomere organisation (*P* = 0.022) and not-yet-annotated (Fig. 2a). The latter term consists of functionally uncharacterised genes and therefore does not provide any insights into the function of this lincRNA. Furthermore, *SUT042* also showed a specific profile since its screen enriched GO terms (membrane fusion *P* = 0.039; organelle fusion *P* = 0.020; vesicle organisation *P* = 0.020) associated with vesicle function (Fig. 2a). On the other hand, the other three lincRNAs, *SUT014*, *SUT451* and *SUT469*, exhibited GO profiles that are more diverse in function. *SUT451* and *SUT469* SGA screens each enriched seven different biological processes, while *SUT014* showed the most varied profile with enrichment of ten different GO terms (Fig. 2a). The heterogeneous GO term profiles suggest that the latter three SUTs are pleiotropic or that the enriched cellular processes are interconnected [43]. Altogether, these results indicate that, under physiological conditions, intergenic SUTs are implicated in a broad spectrum of biological processes that are important for normal cell growth.

### *SUT457* and *SUT042* exhibit distinct GI profiles in comparison to their adjacent genes

Previously characterised SUTs and other yeast non-coding RNAs have been linked to transcriptional regulation of their neighbouring genes [2]. Therefore, we reasoned that SGA analysis of lincRNAs, which control their cognate genes, will lead to the discovery of GIs which would be similar to those identified for their neighbouring genes. To compare the GI networks of the examined intergenic SUTs with those of their neighbouring genes, we exploited the GI data of protein-coding genes available on the DRYGIN dataset (http://drygin.ccbr.utoronto.ca/; accessed 29 Feb 2016) and those generated by Szappanos et al*.* [53], which are compatible with the SGA approach used in this study. We first compared the GO enrichment profiles of *TLC1* with its upstream and downstream gene, *PDX3* and *CSG2*, respectively, since *TLC1* is not linked to the transcriptional control of its neighbours. As predicted, the GIs of *TLC1* enrich GO terms that are distinct to those enriched by the GIs of its flanking genes (Fig. 2b). Similarly to *TLC1*, two other lincRNAs, *SUT457* and *SUT042*, exhibit GO enrichment profiles that are completely different from those of their upstream and downstream neighbouring genes (Fig. 2c and Additional file 7: Figure S2). In contrast, *SUT014*, *SUT451* and *SUT469*, whose GIs enrich diverse biological processes, display GO profiles that partially overlap those of their adjacent genes (Additional file 7: Figure S2). These findings suggest that *SUT014*, *SUT451* and *SUT469* might have functions that are linked to the expression of their neighbouring genes, while *SUT457* and *SUT042* have roles that are independent to the function of their adjacent genes.

### Deletion of *SUT457* accelerates senescence in telomerase-deficient cells

**Fig. 3:**
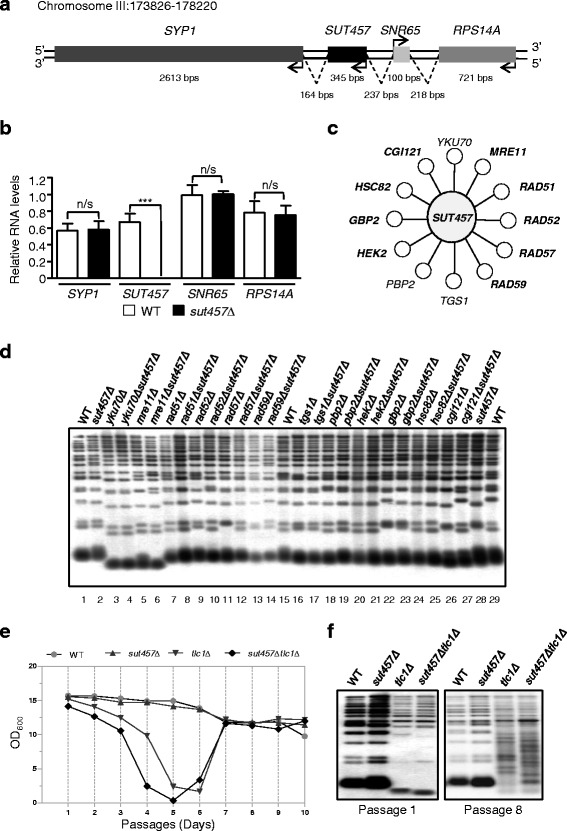
Deletion of *SUT457* accelerates senescence in telomerase-deficient cells. **a** Schematic representation of the *SUT457* locus on Chromosome III. Arrows indicate direction of transcription. **b** Expression levels of *SYP1*, *SUT457*, *SNR65* and *RPS14A* determined by qRT-PCR using total RNA extracted from isogenic wild-type and *sut457Δ* strains. The expression levels of each gene were normalised to the expression of *ACT1*. Error bars represent standard error of the mean, resulting from three independent replicates. *** *P* < 0.001; n/s, not significant (generated by t-test). **c** Genetic interactions of *SUT457* associated with the gene ontology term telomere organisation. Gene names in bold represent negative genetic interactions while gene names in regular font correspond to positive genetic interactions. **d** Telomere length analysis of genomic DNA isolated from the indicated wild-type and mutant strains at passage 1. The extracted DNA was fragmented with *XhoI* and subjected to denatured southern blotting using a biotinylated probe against telomeric repeats. **e** Senescence assays performed using liquid cultures of the indicated isogenic wild-type and mutant strains. The strains were generated through tetrad dissection of the heterozygous diploid double mutant *SUT457/sut457ΔTLC1/tlc1Δ*. This plot represents one of three independent tetrads examined (see also Additional file 9: Figure S4). **f** Telomere length analysis performed as in (**d**) using genomic DNA isolated from the indicated wild-type and mutant strains during passages 1 and 8

*SUT457* showed a very specific GO enrichment profile that was distinct from its flanking genes (Fig. 2) and, therefore, we decided to further characterise its cellular role. Initially, we verified by northern blot analysis the loss of SUT457 transcript (345 bp) in the *sut457Δ* strain which was processed in the SGA screens (Additional file 8: Figure S3). Then, we examined whether construction of the *sut457Δ* strain genetically perturbs the expression of its neighbouring genes (Fig. 3a). Quantitative RT-PCR analysis showed that in *sut457Δ* cells the expression of *SUT457*-adjacent genes *SYP1*, *SNR65* and *RPS14A* remains unaffected compared to a wild-type control strain (Fig. 3b). This result demonstrates that construction of *SUT457* deletion does not lead to the previously reported neighbouring gene effect [54, 55] and, hence, it confirms that the GI network of *sut457Δ* is directly associated with *SUT457* itself and not with its adjacent genes.

SUT457 genetically interacts with 12 out of 42 telomere organisation genes (*P* = 0.0145) found in the SGA deletion collection (Fig. 3c). These genes are associated with roles in supporting telomerase function (*HEK2*, *PBP2*, *YKU70*, *TGS1*, *HSC82*) [56–59], modulating subtelomeric chromatin changes (*PBP2* and *HEK2*) [56], regulating telomere-end processing (*YKU70*, *GBP2*, *MRE11*, *CGI121*, *TGS1* and *HSC82*) [37, 58, 60–63], and controlling telomere maintenance via recombination (*RAD51*, *RAD52*, *RAD57*, *RAD59*, *MRE11*, *CGI121*) [33, 34]. The genetic link between *SUT457* and telomere organisation initially prompted us to determine if loss of this lincRNA leads to changes in telomere length. To do this, we used denaturing southern analysis and found that deletion of *SUT457* alone does not affect telomere length compared to an isogenic wild-type strain (Fig. 3d, compare lanes 1 and 2, 28 and 29). Then, we examined whether *sut457Δ*, in combination with deletion of any of its 12 genetic interactors (Fig. 3c), affects telomere length. As expected, significantly shorter telomeres were detected in the *yku70Δ* and *mre11Δ* single mutants compared to wild-type cells, but telomere length was not further affected in the *yku70Δsut457Δ* or *mre11Δsut457Δ* double mutants (Fig. 3d, compare lanes 3 and 4, 5 and 6). Consistently, *sut457Δ*, in combination with deletion of the remaining telomere-related genetic interactors (Fig. 3c), does not have a significant change in telomere length compared to its isogenic single deletion mutants (Fig. 3d, lanes 7–14 and 16–27).

Based on the fact that some of the telomere-associated GIs of *SUT457* (e.g. *RAD51*, *RAD52*, *RAD57*, *MRE11*) are known to affect entry into senescence in telomerase-deficient cells [34, 52], we next sought to determine if deletion of *SUT457* influences the rate of senescence in *TLC1* null cells. Therefore, we performed liquid senescence assays using the four isogenic strains, namely wild-type, *sut457Δ*, *tlc1Δ* and the double mutant *sut457Δtlc1Δ*. Consistent with the fact that single deletion of *SUT457* had wild-type telomeric length (Fig. 3d), we observed that *sut457Δ* and wild-type strains had a very similar growth profile in senescence assays (Fig. 3e and Additional file 9: Figure S4). However, we found that the double mutant *sut457Δtlc1Δ* had an accelerated entry into senescence compared to the *tlc1Δ* single mutant (Fig. 3e and Additional file 9: Figure S4). Interestingly, this faster induction of senescence correlates with the appearance of shorter telomeres in the *sut457Δtlc1Δ* double deletion strain compared to the *tlc1Δ* single mutant (Fig. 3f, left panel). Moreover, deletion of *SUT457* did not affect the generation of Type II survivors in strains lacking telomerase (Fig. 3f, right panel). Altogether, these findings link the function of *SUT457* to telomere organisation and specifically show that *SUT457* participates within a telomerase-independent pathway.

### Loss of *SUT457* leads to accumulation of telomeric single-stranded DNA

**Fig. 4:**
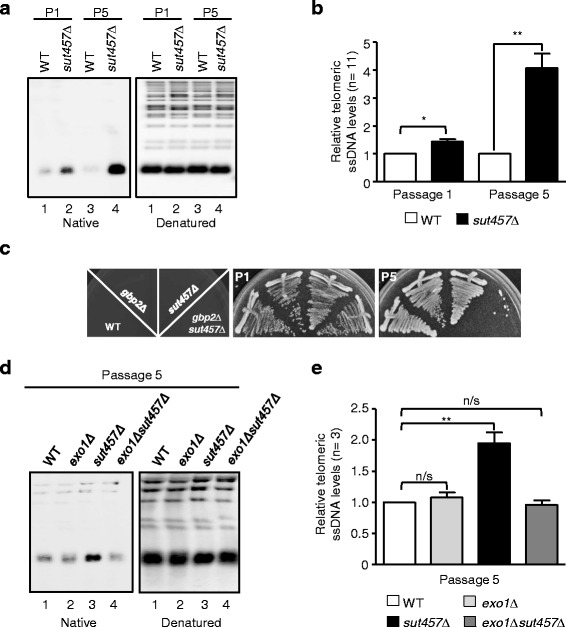
Loss of *SUT457* leads to Exo1-dependent accumulation of telomeric ssDNA. **a** Analysis of telomeric ssDNA overhangs in isogenic wild-type and *sut457Δ* strains. Yeast colonies were restreaked on agar plates and genomic DNA was extracted at passages 1 (lanes 1 and 2) and 5 (lanes 3 and 4), fragmented with *XhoI* and subjected to native southern blotting (left panel) using a biotinylated probe against telomeric repeats. The southern blot was then treated with 0.4 N NaOH and the denatured DNA was re-probed to monitor equal loading (right panel). **b** Accumulation of telomeric ssDNA in eleven *sut457Δ* strains compared to their respective wild-type strains during passages 1 and 5 (P1 & P5). The ssDNA signal for each wild-type and mutant strain detected in the native southern blot was normalised to the corresponding signal in the denatured southern blot. Error bars represent standard error of the mean. * *P* < 0.05; ** *P* < 0.01; n/s, not significant (generated by nonparmetric one-tailed Mann–Whitney t-test). **c** Cell growth was monitored for the indicated isogenic strains at passages 1 and 5. **d** Analysis of telomeric ssDNA levels at passage 5 of wild-type, *sut457Δ*, *exo1Δ* and *exo1Δsut457Δ* isogenic strains generated through dissection of the heterozygous diploid double mutant *EXO1/exo1ΔSUT457/sut457Δ*. Southern blotting was performed as in (**a**) above. **e** Telomeric ssDNA levels analysed at passage 5 in the indicated isogenic wild-type and mutant strains generated from three independent tetrads (*n* = 3). The quantification of the ssDNA signal for each wild-type and mutant strain was performed as in (**b**) above. Error bars represent standard error of the mean. ** *P* < 0.01; n/s, not significant. The statistical analysis was performed using one-way ANOVA (Dunnett’s test)

It has been previously reported that faster senescence in telomerase-negative cells can be attributed to abnormal accumulation of telomeric ssDNA [40, 41]. Thus, we hypothesised that deletion of *SUT457* may lead to the accumulation of ssDNA at telomeres. This hypothesis was further supported by the SGA findings above, which show that *SUT457* genetically interacts with the genes *YKU70*, *MRE11*, *TGS1*, *CGI121*, *HSC82* and *GBP2* (Fig. 3c) that encode for factors implicated in telomere-end protection and in the formation of telomeric ssDNA overhang [35]. To examine this hypothesis, we extracted genomic DNA from isogenic wild-type and *sut457Δ* cells at an early passage and performed native southern blotting using a C-rich oligonucleotide probe. In addition, we extracted genomic DNA from a later passage to mimic the subculturing of cells resulting from the serial pinning steps performed during the SGA procedure. Absence of *SUT457* led to an increase in the signal of telomeric ssDNA compared to wild-type cells in passage 1 (P1), which became even more intense after subculturing yeast cells for five passages (P5) (Fig. 4a). In order to verify that the detected ssDNA signal is due to an increase in 3’ terminal overhang and not due to accumulation of internal DNA replication intermediates, we treated the native DNA with bacterial Exonuclease I that can only degrade terminal ssDNA in a 3’ to 5’ direction. The single stranded DNA that accumulated in *sut457Δ* mutant was sensitive to bacterial Exonuclease I digestion (Additional file 10: Figure S5), indicating that the detected ssDNA indeed corresponds to telomeric 3’ overhang. We also validated the increase in telomeric overhang by comparing the levels of ssDNA among 11 different *sut457Δ* mutant clones and their corresponding wild-type strains. As above, we observed statistically significant accumulation of telomeric ssDNA in *sut457Δ* strains at an early passage (P1) which became even more substantial at a later passage (P5) (Fig. 4b).

Accumulation of telomeric ssDNA has been proposed to be a signal for cell cycle arrest [39]. However, we did not observe a growth reduction in the *sut457Δ* mutant strain compared to wild-type cells (Figs. 3e and 4c). It was previously demonstrated that growth arrest associated with telomeric overhang accumulation is masked by telomeric ssDNA binding proteins like Gbp2 [60]. Interestingly, *GBP2* was identified as a *SUT457* NGI (Fig. 3c) and, therefore, we next examined how lack of Gbp2 can affect the growth of *sut457Δ* cells. We observed a strong growth arrest of *gbp2Δsut457Δ* cells at passage 5 (Fig. 4c), which coincides with the robust accumulation of telomeric ssDNA (Figs. 4a, b). Altogether, these data suggest that *SUT457* controls the levels of telomeric ssDNA overhang in order to maintain proper cellular growth.

### Exo1 nuclease is required for the accumulation of telomeric ssDNA in *sut457Δ* cells

The 5’–3’ exonuclease Exo1 plays a key role in telomere G-rich overhang formation in yeast by mediating C-rich strand degradation [64]. The action of Exo1 is blocked by the Ku complex since lack of Yku70 leads to unprotected telomere ends and results in Exo1-dependent accumulation of telomeric ssDNA [37, 38, 65]. Because *SUT457* genetically interacts with *YKU70* (Fig. 3c), we hypothesised that Exo1 activity may be responsible for the accumulation of telomeric overhang in *sut457Δ* cells*.* To test this hypothesis, we wanted to generate an *exo1Δsut457Δ* double mutant strain by crossing the *exo1Δ* and *sut457Δ* single mutants, but we realised that *EXO1* was not properly deleted in the *exo1Δ* strain found in the SGA knockout library. Therefore, we constructed a new *exo1Δ* deletion strain, mated it to the *sut457Δ* mutant and generated isogenic haploid strains that were analysed for telomeric ssDNA levels. Consistent with the above findings, we detected a strong signal in *sut457Δ* corresponding to the accumulation of telomeric ssDNA after subculturing yeast for 5 passages (Fig. 4d). Notably, the telomeric overhang signal in the *exo1Δsut457Δ* double mutant cells is reduced back to wild-type levels (Fig. 4d, compare lanes 1, 3 and 4). This result was verified by quantifying the levels of telomeric ssDNA in isogenic mutant strains generated from three independent tetrads (Fig. 4e). Furthermore, deletion of *EXO1* in *gbp2Δsut457Δ* cells rescued their growth arrest at passage 5 suggesting that *exo1Δ* suppresses the effect of *sut457Δ* (Additional file 11: Figure S6). Altogether, these findings show that *SUT457* functions within an Exo1-dependent pathway to affect telomeric overhang accumulation.

### The lincRNA SUT457 acts in *trans* to regulate the levels of telomeric ssDNA

**Fig. 5:**
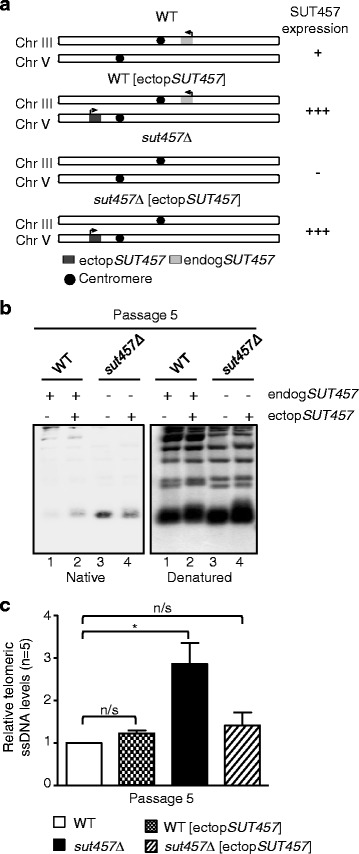
SUT457 regulates the levels of telomeric ssDNA by acting in *trans*. **a** Schematic illustration indicating the presence of endogenous (endog*SUT457*, light grey box) and ectopic *SUT457* (ectop*SUT457*, dark grey box) in wild-type and *sut457Δ* strains. The level of *SUT457* expression for each constructed strain is indicated on the right: + (wild-type levels of expression), +++ (higher expression than wild-type), and – (no expression). Arrows indicate the direction of SUT457 transcription at each locus. **b** Analysis of telomeric ssDNA levels in wild-type (lane 1), wild-type [ectop*SUT457*] (lane 2), *sut457Δ* (lane 3), and *sut457Δ* [ectop*SUT457*] (lane 4) strains at passage 5. Genomic DNA was extracted, fragmented with *XhoI* and subjected to native southern blotting using a biotinylated probe against telomeric repeats (left panel). The southern blot was then treated with 0.4 N NaOH and the denatured DNA was re-probed to monitor equal loading (right panel). **c** Telomeric ssDNA levels were analysed in five independent clones of the indicated wild-type and mutant strains at passage 5. The ssDNA signal for each strain detected in the native southern blot was normalised to the corresponding signal in the denatured southern blot. Error bars represent standard error of the mean. * *P* < 0.05; n/s, not significant. Statistical analysis was performed using one-way ANOVA (Dunnett’s test)

The GI profile of *SUT457* was distinct from that of its adjacent genes (Fig. 2c) and this is also consistent with the fact that loss of *SUT457* does not affect the expression of its neighbours (Fig. 3b). Therefore, we hypothesised that, unlike most characterised yeast lncRNAs, which control the expression of their cognate genes, SUT457 may function in *trans* distant from its locus of synthesis. To address this hypothesis, we examined whether ectopic expression of *SUT457* could rescue the phenotype of telomeric overhang accumulation observed in *sut457Δ* cells (Fig. 4a). For this purpose, we deleted SUT457 from its endogenous locus on chromosome III and re-introduced it on chromosome V under the control of the URA3 promoter (Fig. 5a, *sut457Δ* [ectop*SUT457*]). We also constructed another strain in which SUT457 was ectopically expressed at the URA3 locus in wild-type cells to serve as an additional control (Fig. 5a, wild-type [ectop*SUT457*]). Native southern blot analysis indicated that deletion of *sut457Δ* from its endogenous locus results in robust increase in telomeric ssDNA at passage 5 as previously shown (Figs. 4 and 5b, compare lanes 1 and 3), but concomitant ectopic expression of SUT457 reduces the telomeric overhang signal to corresponding wild-type levels (Fig. 5b). This result was verified by quantifying the levels of telomeric ssDNA in five independent constructed strains (Fig. 5c). Collectively, these findings demonstrate that SUT457 can control the levels of telomeric ssDNA overhang regardless of its genomic location, indicating that this lincRNA functions in *trans*.

## Discussion

Despite the plethora of long non-coding RNAs identified by genome-wide approaches, rigorous genetic studies that could aid in the functional characterisation of individual lncRNAs are missing [2]. Here, we took advantage of the genetic tractability of yeast and employed a high-throughput SGA methodology to systematically catalogue the GIs of six intergenic SUTs. Initially, we verified the utility of the approach by demonstrating that GIs identified for the telomerase RNA *TLC1* enrich GO terms corresponding to its known cellular function. Subsequent SGA screens and analysis of GI profiles implicated the six tested intergenic SUTs in diverse biological processes. One of these lincRNAs, *SUT457*, exhibited GIs that enriched a definitive GO annotation linking it to telomere biology. Further functional characterisation unveiled the role of *SUT457* in preventing the accumulation of telomeric ssDNA. Moreover, phenotypic rescue experiments showed that SUT457 acts in *trans* to maintain physiological levels of telomeric ssDNA. Our work reveals that systematic mapping of the GIs of lncRNAs could associate these molecules with specific biological processes and pinpoint their individual functions.

In yeast, the function of identified lncRNAs has been mainly linked to the regulation of gene expression. Most lncRNAs characterised so far regulate their cognate genes either through transcriptional interference [23, 24, 66] or by modulating their local chromatin structure [19–22]. However, emerging evidence suggest that lncRNAs could have roles beyond gene regulation [42]. The GO enrichment profiles of the tested intergenic SUTs have linked them to nuclear functions such as DNA repair, meiosis and telomere organisation but also to cytoplasmic processes including membrane fusions and vesicle formation, for example, in the case of *SUT042* (Fig. 2a). The latter observation is consistent with the fact that a large number of SUTs are transported to the cytoplasm and have been proposed to represent functional transcripts within this cell compartment [18]. Moreover, human lncRNAs are co-expressed with cytoplasmic membrane proteins [67] and are present within extracellular vesicles [68]. Therefore, future in-depth characterisation of lincRNAs, like *SUT042*, could elucidate the role of these molecules in cytoplasmic processes such as vesicle biogenesis and trafficking.

Only rare examples of *trans*-acting yeast lncRNAs have been reported to date. Specifically, the *PHO84* antisense lncRNAs and a cryptic unstable transcript associated with Ty1 retrotransposons control transcriptional silencing of their target genes in *trans* [69, 70]. We show here that GI profiling could also provide insights about the mode of action of lincRNAs in addition to inferring their biological function. This is achieved by comparing the GI profiles of lincRNAs with those of their adjacent protein-coding genes. For example, *TLC1* has a GI network that completely differs from that of its upstream and downstream genes (Fig. 2b) which is in line with the fact that *TLC1* has a function unrelated to its neighbours and acts in *trans* at chromosome ends [71]. Similarly, *SUT457* and *SUT042* have distinct GO profiles compared to their neighbouring genes (Fig. 2c and Additional file 7: Figure S2), suggesting that these SUTs may also work in *trans*. Accordingly, *SUT457* deletion does not affect the expression of its neighbouring genes (Fig. 3b) and regulates a telomeric process occurring distal from its transcriptional site (Fig. 5). However, there is still a possibility that the *SUT457* locus is proximal to a gene involved in telomere-end processing within the three-dimensional architecture of the genome. Hence, in such a scenario, *SUT457* would have a role in telomere-end processing by controlling its spatially proximal gene. Nevertheless, the phenotypic rescue obtained by expressing *SUT457* from a different chromosome (Fig. 5) makes this scenario rather unlikely. Three of the other intergenic SUTs tested (*SUT014*, *SUT451* and *SUT469)* exhibit GIs with GO enrichment profiles that overlap those of their flanking genes (Additional file 7: Figure S2), indicating that these lincRNAs may be involved in the regulation of their nearby genes in *cis*. Of course, we cannot eliminate, at this point, the likelihood that construction of these three SUT deletions leads to a neighbouring gene effect [54, 55], which might be responsible for the observed overlap among enriched GO profiles (Additional file 7: Figure S2). We note that two of the six tested intergenic SUTs (*SUT042* and *SUT457*) exhibit GI profiles consistent with a *trans*-acting role and, therefore, in contrast to current evidence in the literature, we anticipate that a considerable number of budding yeast lincRNAs would function at sites distal from their locus of synthesis.

Lack of *SUT457* exhibited accelerated senescence in telomerase-negative cells (Fig. 3e and Additional file 9: Figure S4) associated with enhanced telomere shortening (Fig. 3f) and an accumulation in telomeric ssDNA overhang (Fig. 4a). Interestingly, analogous phenotypes have been reported for mutations of factors involved in telomere-end protection [38, 40, 41, 52]. Consistent with the above observations, *SUT457* shows a positive GI with the telomere-capping factor *YKU70* (Fig. 3c and Additional file 12: Figure S7), implying that these two molecules affect the same molecular process [46]. Notably, Yku70 binds to and protects telomeres from Exo1 nucleolytic processing, thus preventing the accumulation of telomeric ssDNA overhang [30, 37, 64]. Our data also show that the Exo1 nuclease is required for the increase in telomeric ssDNA detected in the absence of SUT457 (Figs. 4d and e). Furthermore, in agreement with the fact that *EXO1*-dependent accumulation of telomeric ssDNA induces cell-cycle arrest [37, 72], we show that loss of the telomeric ssDNA masking protein Gbp2 in *sut457Δ* cells results in growth inhibition after subculturing (Fig. 4c) and, importantly, this growth arrest is rescued by deletion of *EXO1* (Additional file 11: Figure S6). Based on the above evidence, we speculate that SUT457 functions in a pathway that protects telomere-ends from nucleolytic processing in order to regulate the levels of telomeric ssDNA overhangs. Interestingly, the telomere-associated lncRNA telomeric repeat-containing RNA has also been implicated in this pathway but instead facilitates the nuclease activity of Exo1 at chromosome ends [73].

The primary nucleotide sequence of *SUT457* (Additional file 6: Table S5) does not display significant sequence similarity with any other regions within the *S. cerevisiae* genome and lacks conservation even amongst closely related yeast species ([11] and data not shown). Nevertheless, considering its unveiled role in the fundamental and conserved cellular process of telomere overhang homeostasis [35], we believe that functional analogues of SUT457 may exist in other eukaryotes. Likewise, TLC1 is a lncRNA whose primary sequence is not evolutionarily conserved but which has functional analogues in other organisms [26].

## Conclusions

Transcriptome studies have enabled the discovery of a large number of lncRNAs in various species. However, it remains unclear how many of these lncRNAs serve a biological function or are a mere result of transcriptional noise [74, 75]. We show here that systematic analysis of pairwise GIs can provide insights about the function of individual lncRNAs. The functional maps obtained by GIs could complement the information derived by other high-throughput approaches [76, 77] to help unravel the biological significance of lncRNAs. Although the present study focuses on stable lincRNAs, future work could also analyse GIs for yeast antisense lncRNAs since methodologies are being developed that abrogate the transcription of a specific non-coding RNA without disrupting the expression of its associated sense mRNA [78]. Furthermore, global mapping of GIs for lncRNAs can be conceivably extended to complex organisms because recent advances in RNAi and CRISPR/Cas technologies allow for efficient pairwise gene disruptions in human cells [79–82]. Such analyses may unveil interspecies conservation of GI maps [83] that could shed light on the evolution and functional conservation of lncRNAs [84, 85].

## Methods

### Yeast strains and plasmids

Yeast strains used in this study are described in Additional file 13: Table S6. SGA query strains were constructed by substituting the *TLC1*, *EST1* or a candidate SUT locus with the NatMX4 cassette, which confers cloNAT (Nourseothricin) antibiotic resistance in Y7092 background strain (*MATalpha can1Δ::STE2pr-Sp_his5 lyp1Δ his3Δ1 leu2Δ0 ura3Δ0 met15Δ0*). The PCR fragment used for the above deletions was generated by primers listed in Additional file 14: Table S7. The SGA library consists of 4309 BY4741 (MATa his3*Δ*1 leu2*Δ*0 ura3*Δ*0 met15*Δ*0) single knockout strains with each one carrying deletion of a non-essential gene. The genes were replaced with the antibiotic marker KanMX4, which confers resistance to G418 (Geneticin). The *exo1Δ* strain was constructed from scratch by transforming BY4741 cells with a PCR fragment that replaced the *EXO1* Open Reading Frame with the KanMX4 cassette. The Y8835 strain in which the *HIS3* gene is substituted by the NatMX4 cassette is used as the query strain in control SGA screens [44].

All isogenic strains used for follow-up experiments were generated by crossing the *sut457Δ* single mutant with single knockout strains of interest. Diploid cells were sporulated on 2% potassium acetate for 7 days at room temperature. Tetrads were isolated and subsequently manipulated using a dissection microscope (SporePlay, Singer Instruments) to generate isogenic wild-type, single and double mutant strains. All genotypes were verified by PCR analysis. For the construction of strains ectopically expressing SUT457, isogenic wild-type and *sut457Δ* cells were subcultured to passage 5 and then transformed with a PCR fragment containing the *SUT457* sequence. The PCR fragment was integrated on chromosome V by homologous recombination in front of the *URA3* promoter. The ectopic expression of SUT457 was verified by quantitative real-time PCR (qRT-PCR).

### Selection of intergenic SUTs

To select intergenic SUTs, we developed an in-house perl script (filter-SUTs-2.pl) that identified, among all SUTs listed in the supplementary material of Xu et al. [11], the ones that did not overlap any of the genomic features (file SGD_features.tab, date stamp 20070811, listed in Additional file 5: Table S4) annotated in the SGD database (http://www.yeastgenome.org; accessed: Jan 2013). For any given SUT, genomic elements in both strands were taken into account. The list of selected intergenic SUTs is shown as Additional file 4: Table S3.

### Synthetic genetic array (SGA)

The knockout query strain harbouring the deletion of interest (Y7092), in this case of a lincRNA or *EST1* gene, was crossed against the SGA single deletion library described above. The diploid cells were sporulated, germinated and passaged as previously described [44], using a BM3-BC colony processing robot (S&P Robotis Inc.). Haploid mutants containing the deletion of interest (lincRNA or *EST1*) and/or the corresponding protein-coding gene deletion were isolated as described previously [44], apart from the following modifications in the protocol in order to improve population purity: (1) strains were pinned two times instead of one on media selecting for *can1Δ*, *lyp1Δ* and STE2pr-Sp_his5, and (2) strains were pinned two times instead of one on media selecting for double deletions (Fig. 1a). The same procedure was followed in parallel SGA screens using the control query strain (Y8835).

Quantification of yeast colony size on the final selection plates of the SGA screen was accomplished using spImager (S&P robotics Inc., Toronto, Canada). For each plate, we carried out a normalisation procedure (using a custom built perl script) based on the average colony growth detected on the specific plate, in order to correct for uneven plate growth. We excluded from further analysis: (1) colonies on the periphery of the plate (YOR202W), (2) genes for which the single deletion strain harbouring the relative library gene deletion grew less than 60% of the average growth, and (3) linkage group loci (composed of 30 genes upstream and downstream from the query gene of interest) to avoid artefacts related to potential linkage disequilibrium [44]. Finally, we report, as potential GIs, those genes for which the double deletion strain has at least a ±30% change in growth fitness compared to the single deletion strain. This threshold was defined based on growth changes observed for known *TLC1* GIs.

### Gene ontology (GO) analysis

The genes corresponding to the NGIs identified in each SGA screen were grouped into their annotated GO biological processes according to the Saccharomyces Genome Database (SGD) GO Slim Mapper (http://www.yeastgenome.org/cgi-bin/GO/goSlimMapper.pl). A comprehensive list of all 102 GO biological processes used during this analysis is provided in Additional file 15: Table S8. The Fisher exact test (significance level α = 0.05) was used to identify statistically significantly enriched GO terms. The same GO analysis was performed on NGI datasets generated in this work and NGIs obtained from previous studies.

### Venn diagrams

Venn diagrams for GI datasets were generated using the on-line tool Venny 2.0 (http://bioinfogp.cnb.csic.es/tools/venny/). Significance of the overlap between gene sets was evaluated using the hypergeometric test, as implemented in the R function ‘phyper’ (http://www.R-project.org/), setting the significance level to α = 0.05.

### RNA isolation and gene expression analysis

Total RNA from logarithmically grown (OD_600_ of 0.8) yeast cells was isolated using the hot phenol extraction method [86] and treated with the TURBO DNA-free DNase kit (Ambion, AM1907). For cDNA preparation, 0.5 μg RNA (DNase treated) was treated with PrimeScript Reverse Transcriptase (Takara, 2680A). The quantity of cDNA was determined by qRT-PCR analysis performed on a Bio-Rad CFX96 Real-Time PCR system using SYBR Green (Kapa SYBR Fast Master Mix # KK4602) and the primers listed in Additional file 14: Table S7.

### Northern blot analysis

Isolated total RNA was first treated with DNAse and then mixed with 10 μL deionised formamide, 3.5 μL 37% formaldehyde and 2 μL loading buffer (0.1 M MOPS pH 7, 40 mM sodium acetate, 5 mM EDTA pH8.0) to a final volume of 20 μL. The sample was then heated at 70 °C for 10 min and electrophoresed on agarose-formaldehyde gel (1% agarose, 200 mM MOPS pH 7, 10 mM EDTA, 50 mM NaOAC, 6.7% formaldehyde) at 100 volts for 30 minutes. The gel was stained with ethidium bromide (0.75 μg/mL) to ensure RNA integrity and then equilibrated with 10× SSC buffer for 30 min (150 mM NaCl and 15 mM sodium citrate, pH 7) prior to transferring onto Hybond-N + membrane (GE Healthcare, RPN303B) using overnight capillary transfer with 20× SSC. The RNA was UV cross-linked (700 Joules/cm^2^) onto the membrane and hybridised with 50 ng/mL biotinylated probes (Additional file 14: Table S7) in 25 mL Church buffer (0.5 M NaPO_4_ pH 7.2, 1 mM EDTA pH 8, 7% SDS, 1% BSA) at 50 °C overnight. The membrane was treated with Chemiluminescent Nucleic Acid Detection Module (ThermoFisher Scientific, 89880) and then exposed using the UVP Bioimaging system (Syngene).

### Native and denatured southern blotting

Genomic DNA from non-synchronised saturated cell cultures was digested overnight with *XhoI* (Takara, 1094A) and then separated on 1% agarose gel (15 cm in length) for 18 hours at 25 volts. *E. coli* exonuclease I digestion (New England biolabs, M0293S) was performed prior to *XhoI* digestion of the genomic DNA. Analysis of the telomeric single-stranded overhangs was performed under native conditions by treating the agarose gel with 10× SSC buffer (0.17 M trisodium citrate, 1.5 M NaCl) for 30 minutes at room temperature. The DNA bands were then transferred onto Hybond-N + membrane (GE Healthcare, RPN303B) using a Trans-Blot Semi-Dry electrophoretic transfer cell (Bio-Rad Laboratories) as previously described [87]. The DNA was UV cross-linked (700 Joules/cm^2^) on the membrane and hybridised with 50 ng/mL biotinylated C_1-3_A probe (Additional file 14: Table S7) in 25 mL Church buffer (0.5 M NaPO_4_ pH 7.2, 1 mM EDTA pH 8, 7% SDS, 1% BSA) at 50 °C overnight. Probe-bound DNA fragments corresponding to telomeric single-stranded overhangs were treated with the Chemiluminescent Nucleic Acid Detection Module Kit (ThermoFisher Scientific, 89880) and detected using the UVP Bioimaging system (Syngene). Following detection of the single-stranded telomeric overhang, the blot was incubated under denaturing conditions (0.4 M NaOH, 0.1% SDS) at 45 °C for 30 minutes. The blot was then probed and processed as above to detect denatured telomeric fragments. The intensity of the signals corresponding to native and denatured Y’ telomeric bands were quantified by histogram analysis (Adobe Photoshop CC 2015).

### Single-colony re-streaking assay

Isogenic haploid strains derived from tetrad dissection are streaked on solid YPAD (Yeast extract, Peptone, Adenine hemisulfate, Dextrose) medium and incubated at 30 °C for 2 days until the appearance of single colonies (~25 cell divisions) corresponding to passage 1. Then, individual colonies from each wild-type and mutant strain were sequentially re-streaked on new solid YPAD plates until the appearance of colonies corresponding to passage 5 [34].

### Senescence assay

A single colony from each indicated isogenic haploid strain was used to inoculate YPAD liquid medium and the culture was grown overnight at 30 °C until saturation. The following day, each culture was diluted to OD_600_ of 0.01 in fresh YPAD medium, incubated for 24 hours at 30 °C and the OD_600_ was then determined corresponding to the value of passage 1. The culture was then re-diluted to OD_600_ of 0.01 and the same procedure was repeated for passages 2–10 [34].

## Additional files

Additional file 1: Table S1. Negative genetic interactions of all tested lincRNAs. (XLSX 35 kb)
Additional file 2: Table S2. Positive genetic interactions of all tested lincRNAs. (XLSX 45 kb)
Additional file 3: Figure S1. Overlap between *TLC1* and *EST1* negative genetic interactions. **a** Venn diagram showing the overlap (*P* = 0.001 generated using the hypergeometric test) between genes whose deletions result in negative genetic interactions with *TLC1* in this study and with *EST1* as reported in [43]. GO terms enriched by common negative genetic interactions with *P* < 0.05 (generated using the Fisher exact test) are indicated below the Venn diagram. **b** Same analysis as in (**a**) between negative genetic interactions identified for *TLC1* in this study and those reported for *EST1* in [52]. (PDF 8 kb)
Additional file 4: Table S3. Intergenic SUTs identified in Xu et al. [11]. (XLSX 17 kb)
Additional file 5: Table S4. Genomic features considered for selecting intergenic SUTs. (XLSX 8 kb)
Additional file 6: Table S5. Genomic features of tested intergenic SUTs. (XLSX 11 kb)
Additional file 7: Figure S2. Gene ontology (GO) terms enriched by the negative genetic interactions of SUTs and of their flanking genes. Heatmap of GO terms enriched with *P* ≤ 0.05 (generated using the Fisher exact test) by genes classified as negative genetic interactions for the indicated SUTs and for their corresponding adjacent protein-coding genes. The negative genetic interactions of the flanking protein-coding genes were extracted from the Drygin database (http://drygin.ccbr.utoronto.ca/). (PDF 95 kb)
Additional file 8: Figure S3. Detection of SUT457 RNA. Total RNA was isolated from wild-type and *sut457Δ* cells and analysed by northern blotting using a probe (Additional file 14: Table S7) against the *SUT457* sequence (Additional file 6: Table S5). A band of 345 bp corresponding to the size of SUT457 is detected only in wild-type cells (left panel). A probe against actin (Additional file 14: Table S7) was used as a loading control (right panel). (PDF 26 kb)
Additional file 9: Figure S4. Lack of *SUT457* accelerates senescence in *tlc1Δ* cells. **a, b** Senescence assays performed using liquid cultures of the indicated isogenic wild-type and mutant strains. The strains were generated through tetrad dissection of the heterozygous diploid double mutant *SUT457/sut457ΔTLC1/tlc1Δ*. Each plot represents one of three independent tetrads examined (see also Fig. 3e). (PDF 65 kb)
Additional file 10: Figure S5. Degradation of telomeric ssDNA by bacterial Exonuclease I. Genomic DNA extracted from four individual *sut457Δ* strains at passage five was either untreated (-) or digested with bacterial exonuclease I (+), which degrades telomeric overhang DNA in the 3’ to 5’ direction, before being subjected to native southern analysis using a biotinylated probe against telomeric repeats (upper blot). The DNA on the membrane was then denatured with 0.4 N NaOH and re-probed to monitor equal loading (lower blot). (PDF 48 kb)
Additional file 11: Figure S6. Deletion of *EXO1* rescues the growth arrest of *gbp2Δ sut457Δ* mutant cells*.* Isogenic strains of the indicated genotype (top panels) were streaked repeatedly on solid rich medium and their growth at passage 5 is shown. (PDF 68 kb)
Additional file 12: Figure S7. Growth curve analysis validates the positive genetic interaction between *sut457Δ* and *yku70Δ.* The indicated isogenic strains were diluted to OD_600_ of 0.1 and cultured at 30 °C in rich YPD medium until saturation. The OD_600_ of each strain was measured every hour for a total of 24 hours using Infinite M200 (Tecan Trading AG). Error bars represent SEM of eight independent tetrads. (PDF 52 kb)
Additional file 13: Table S6. Yeast strains used in this study. (XLSX 10 kb)
Additional file 14: Table S7. Primer and probe sequences. (XLSX 10 kb)
Additional file 15: Table S8. List of all analysed Gene Ontology Slim terms. (XLSX 10 kb)
Additional file 16: Supporting data. (XLSX 10 kb)

## Abbreviations

GIs: genetic interactions
GO: gene ontology
lincRNAs: long intergenic non-coding RNAs
NGIs: negative genetic interactions
PGIs: positive genetic interactions
SGA: synthetic genetic array
ssDNA: single stranded DNA
SUTs: stable unannotated transcripts
