# Supplementary material for: Functional characterisation of long intergenic non-coding RNAs through genetic interaction profiling in Saccharomyces cerevisiae
Source: BMC Biol. 2016 Dec 7;14:106. doi: 10.1186/s12915-016-0325-7 (PMC5142380; doi:10.1186/s12915-016-0325-7)
Supplement: Additional file 3: Figure S1. — Overlap between TLC1 and EST1 negative genetic interactions. a Venn diagram showing the overlap (P = 0.001 generated using the hypergeometric test) between genes whose deletions result in negative genetic interactions with TLC1 in this study and with EST1 as reported in [43]. GO terms enriched by common negative genetic interactions with P < 0.05 (generated using the Fisher exact test) are indicated below the Venn diagram. b Same analysis as in (a) between negative genetic interactions identified for TLC1 in this study and those reported for EST1 in [52]. (PDF 8 kb) [file 12915_2016_325_MOESM3_ESM.pdf]

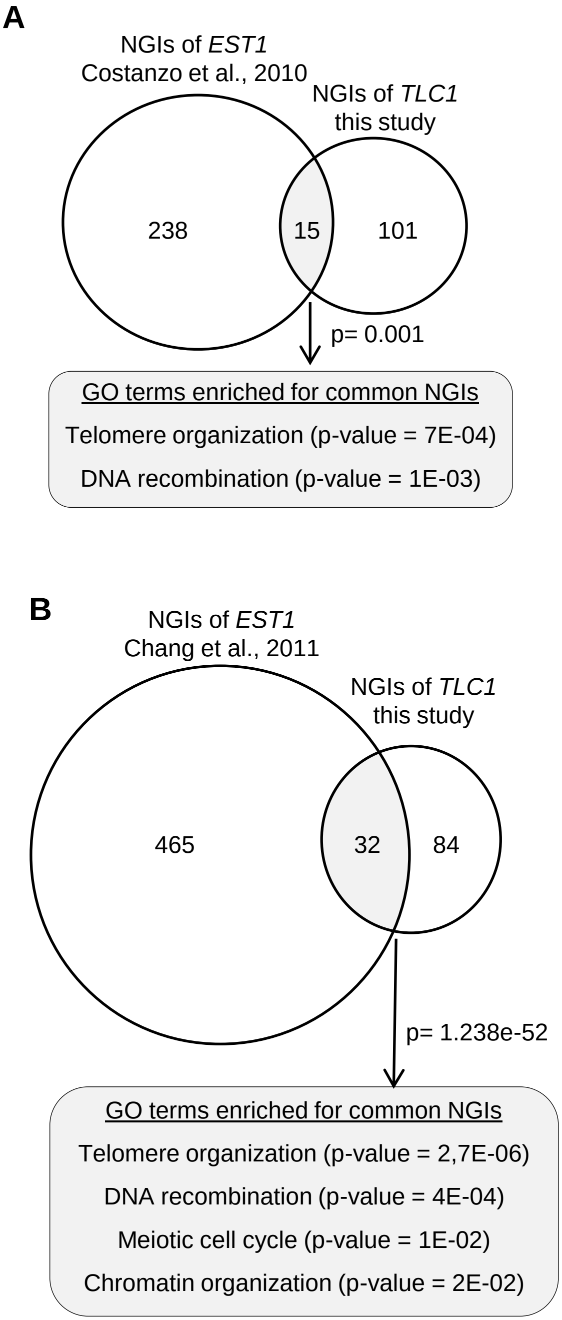
A
NGIs of *EST1*
Costanzo et al., 2010
NGIs of *TLC1*
this study
238
15
101
p= 0.001
GO terms enriched for common NGIs
Telomere organization (p-value = 7E-04)
DNA recombination (p-value = 1E-03)
B
NGIs of *EST1*
Chang et al., 2011
NGIs of *TLC1*
this study
465
32
84
p= 1.238e-52
GO terms enriched for common NGIs
Telomere organization (p-value = 2,7E-06)
DNA recombination (p-value = 4E-04)
Meiotic cell cycle (p-value = 1E-02)
Chromatin organization (p-value = 2E-02)
