# Supplementary figures and images for: Functional characterisation of long intergenic non-coding RNAs through genetic interaction profiling in Saccharomyces cerevisiae
Source: BMC Biol. 2016 Dec 7;14:106. doi: 10.1186/s12915-016-0325-7 (PMC5142380; doi:10.1186/s12915-016-0325-7)

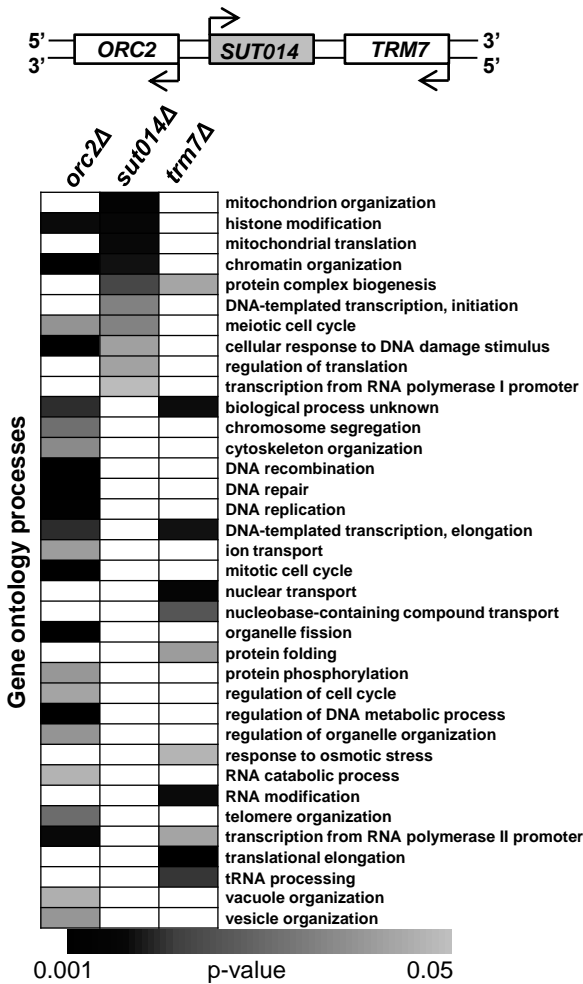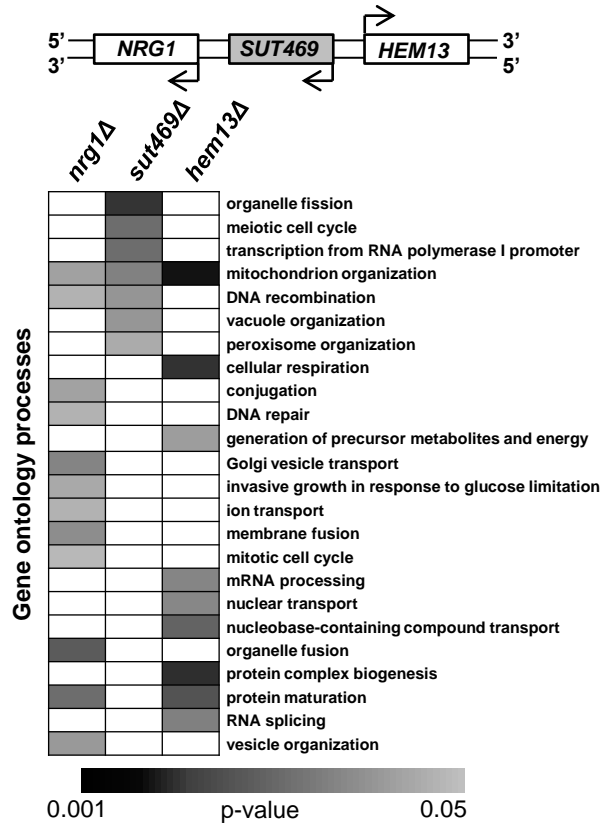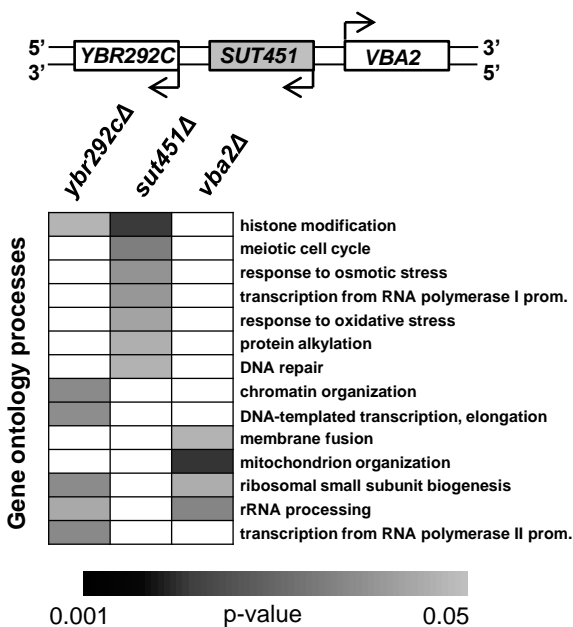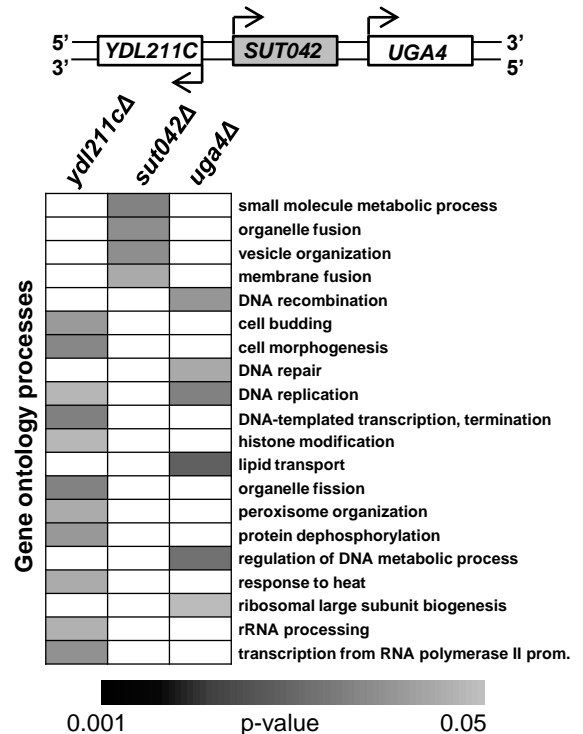

Supplement: Additional file 7: Figure S2. — Gene ontology (GO) terms enriched by the negative genetic interactions of SUTs and of their flanking genes. Heatmap of GO terms enriched with P ≤ 0.05 (generated using the Fisher exact test) by genes classified as negative genetic interactions for the indicated SUTs and for their corresponding adjacent protein-coding genes. The negative genetic interactions of the flanking protein-coding genes were extracted from the Drygin database (http://drygin.ccbr.utoronto.ca/). (PDF 95 kb) [file 12915_2016_325_MOESM7_ESM.pdf]

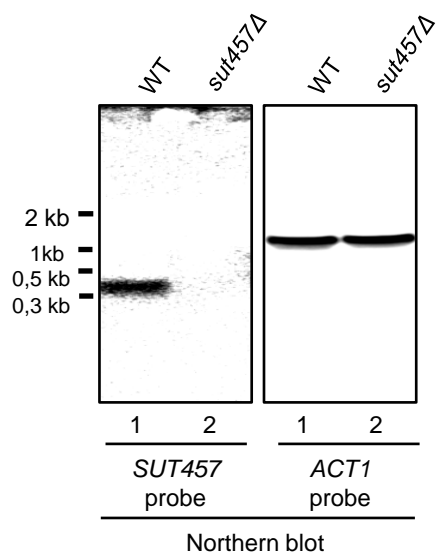

Supplement: Additional file 8: Figure S3. — Detection of SUT457 RNA. Total RNA was isolated from wild-type and sut457Δ cells and analysed by northern blotting using a probe (Additional file 14: Table S7) against the SUT457 sequence (Additional file 6: Table S5). A band of 345 bp corresponding to the size of SUT457 is detected only in wild-type cells (left panel). A probe against actin (Additional file 14: Table S7) was used as a loading control (right panel). (PDF 26 kb) [file 12915_2016_325_MOESM8_ESM.pdf]

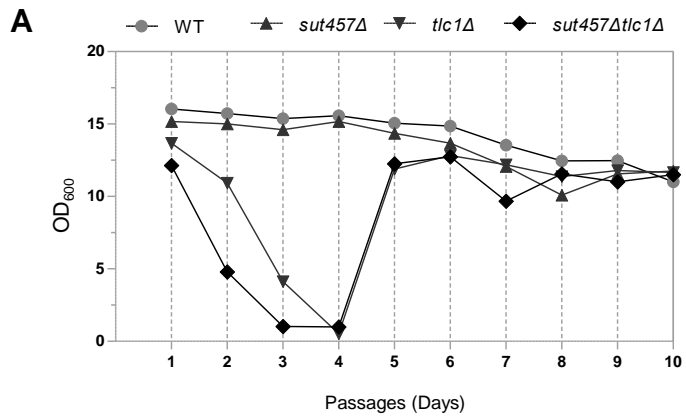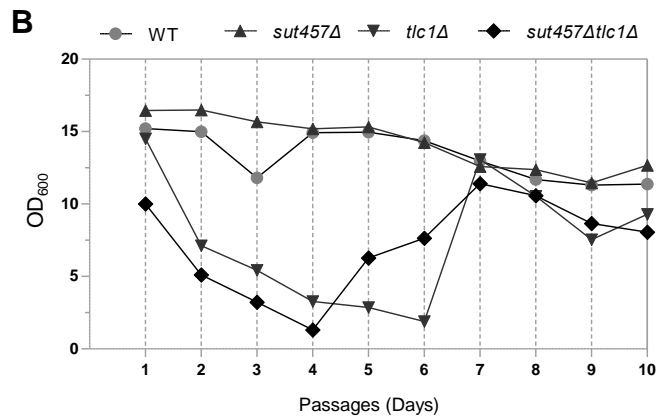

Supplement: Additional file 9: Figure S4. — Lack of SUT457 accelerates senescence in tlc1Δ cells. a, b Senescence assays performed using liquid cultures of the indicated isogenic wild-type and mutant strains. The strains were generated through tetrad dissection of the heterozygous diploid double mutant SUT457/sut457ΔTLC1/tlc1Δ. Each plot represents one of three independent tetrads examined (see also Fig. 3e). (PDF 65 kb) [file 12915_2016_325_MOESM9_ESM.pdf]

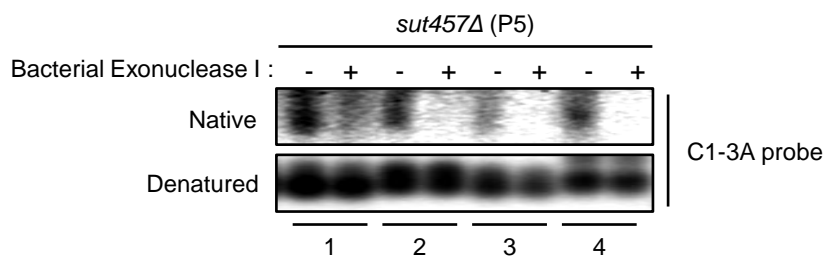

Supplement: Additional file 10: Figure S5. — Degradation of telomeric ssDNA by bacterial Exonuclease I. Genomic DNA extracted from four individual sut457Δ strains at passage five was either untreated (-) or digested with bacterial exonuclease I (+), which degrades telomeric overhang DNA in the 3’ to 5’ direction, before being subjected to native southern analysis using a biotinylated probe against telomeric repeats (upper blot). The DNA on the membrane was then denatured with 0.4 N NaOH and re-probed to monitor equal loading (lower blot). (PDF 48 kb) [file 12915_2016_325_MOESM10_ESM.pdf]

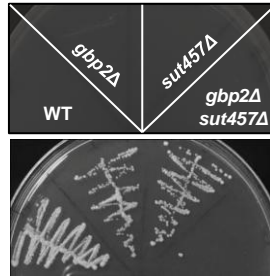

Passage 5

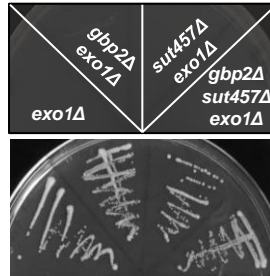

Passage 5

Supplement: Additional file 11: Figure S6. — Deletion of EXO1 rescues the growth arrest of gbp2Δ sut457Δ mutant cells. Isogenic strains of the indicated genotype (top panels) were streaked repeatedly on solid rich medium and their growth at passage 5 is shown. (PDF 68 kb) [file 12915_2016_325_MOESM11_ESM.pdf]

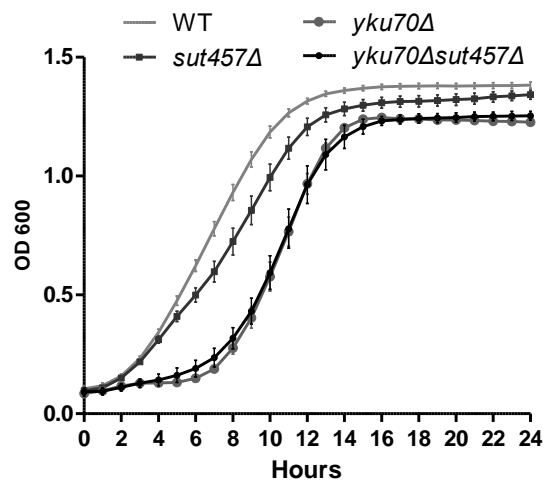

Supplement: Additional file 12: Figure S7. — Growth curve analysis validates the positive genetic interaction between sut457Δ and yku70Δ. The indicated isogenic strains were diluted to OD600 of 0.1 and cultured at 30 °C in rich YPD medium until saturation. The OD600 of each strain was measured every hour for a total of 24 hours using Infinite M200 (Tecan Trading AG). Error bars represent SEM of eight independent tetrads. (PDF 52 kb) [file 12915_2016_325_MOESM12_ESM.pdf]
